# Supplementary material for: A single cell atlas of the mouse seminal vesicle
Source: G3 (Bethesda). 2025 Feb 28;15(5):jkaf045. doi: 10.1093/g3journal/jkaf045 (PMC12060236; doi:10.1093/g3journal/jkaf045)
Supplement: jkaf045_Supplementary_Data [file jkaf045_supplementary_data.zip › Supplemental_Material_Legends_G3-2025-405724.docx]

**SUPPLEMENTAL FIGURES AND TABLES**

**Figures S1-S7**

**Tables S1-S4**

**SUPPLEMENTAL FIGURES**

**Figure S1. Seminal vesicle dissection**

Seminal vesicle from a typical dissection for single cell dissociation. Red arrows on left panel show seminal vesicle in situ, followed by excised seminal vesicle and prostate, and then cleaned seminal vesicle in the rightmost panel.

**Figure S2. Individual samples comprising the full dataset**

a) UMAPs for all 23 samples in the full dataset. “Aging” represents 10 C57Bl/6J samples separately barcoded in a single 10X flowcell, with 2 replicates each of young (3 months) animals, along with 20 month and 28 month animals either raised under control conditions or transiently treated with rapamycin (**Methods**). “Diet” represents 8 FVB/NJ animals raised on the indicated diets (Control, Caloric Restriction, High Fat, Low Protein). Finally, “Individual Samples” refers to 5 individual samples each loaded into a single 10X flow cell.

b-e) Percent mitochondrial reads before and after quality control filtering (panels b and c, respectively), number of cells (d), and number of genes per cell (e), for each of the 23 individual samples in the dataset. Note the much higher number of cells for the five samples run in individual flowcells, relative to the cell counts for each of the 8 and 10 samples barcoded together in the “Aging” and “Diet” flowcells.

**Figure S3. Stromal cell reclustering**

a) UMAP shows reclustered stromal cell populations. Inset shows the full dataset, highlighting the clusters extracted for reclustering. Reclustered cells are annotated according to inferred cell type.

b) Expression of the indicated genes in the reclustered stromal cell UMAP.

**Figure S4. Tgm4 protein and RNA expression in the seminal vesicle**

a-b) TGM4 protein expression in the prostate (a) and seminal vesicle (b). Top panel shows secondary antibody only, bottom shows prostate section stained with anti-TGM4.

c-d) *Tgm4* RNA expression in prostate (c) and seminal vesicle (d). Hybridization chain reaction probes against spliced *Tgm4* mRNA, or against intron-containing pre-mRNA, as indicated.

**Figure S5. Most cytokines detected in seminal fluid are not detectably expressed in seminal vesicle epithelial cells**

UMAPs showing expression of various cytokines previously detected in seminal plasma and linked to seminal fluid signaling in the female reproductive tract. Upper left panel highlights secretory epithelial cells (Epi), macrophages (Mac), and T/NK cells (T) from **Fig. 1a**.

**Figure S6. Robustness of immune cell populations across samples**

a) UMAPs for reclustered immune populations across all 23 samples, showing similar immune populations in 23 distinct animals of two genotypes, from 10 weeks to 28 months of age, and subject to multiple dietary challenges.

b) Overall percentages of immune populations in our dataset. Compare to **Fig. 5a**.

**Figure S7. Flow cytometry gating strategy and representative immune cell populations identified in the murine seminal vesicles.** Seminal vesicle tissue was collected from 8-12 week old adult male Swiss mice and processed for flow cytometry identifying immune cells residing in seminal vesicle tissue.

a) Singlets and intact total cells were selected based on forward scatter (FSC) and side scatter (SSC) followed by live cells (viability dye negative) and leukocytes (CD45+ Ep-CAM- cells).

b) Gating and representative frequencies of macrophages (F480+), natural killer (NK) cells (NKp46+), CD4^+^ T cells (CD4+), CD8^+^ T cells (CD8+), and dendritic cells (DCs; CD11c+ F4/80-).

c) To determine the phenotypes of macrophages present, F4/80+ cells were gated for CD64, CD11c, CD11b and Ly6C expression.

d) Gating for neutrophils (CD11b+ Ly6G+), eosinophils (SiglecF+), and B cells (B220+). Data information: A-D: representative flow cytometry dot plots from one experiment n=4 (biological replicates). B, C: % displayed are the proportions of the parent populations the cells within the gates/quadrants comprise.

**SUPPLEMENTAL TABLES**

**Table S1. Animals used for single cell dataset**

Demographic information, including age and dietary exposures, for the animals used for the seminal vesicle samples throughout this study.

**Table S2. Marker genes for seminal vesicle cell clusters**

Table consists of two worksheets. “Markers” lists significant marker genes for each cluster, identified using the FindAllMarkers function in Seurat with "bimod", likelihood-ratio test for differential gene expression. The full and reproducible workflow is uploaded to github (https://github.com/yufu0012016/SV_Rando), with this analysis located in notebook 3.Label-cluster.ipynb. “Cluster_IDs” provides cell type names for each of the numbered cluster IDs.

**Table S3. Gene expression for cell populations in the murine seminal vesicle**

RNA abundance for each of the major seminal vesicle cell clusters shown in **Fig. 1a**. RNA reads were accumulated for all the cells in a given cluster, and then normalized to a total of 10,000 for each cluster.

**Table S4. Gene expression for immune cell subclustering**

RNA abundance for each of the immune cell clusters shown in **Fig. 4a**. As in **Table S3**.
