# Supplementary figures and images for: A single cell atlas of the mouse seminal vesicle
Source: G3 (Bethesda). 2025 Feb 28;15(5):jkaf045. doi: 10.1093/g3journal/jkaf045 (PMC12060236; doi:10.1093/g3journal/jkaf045)

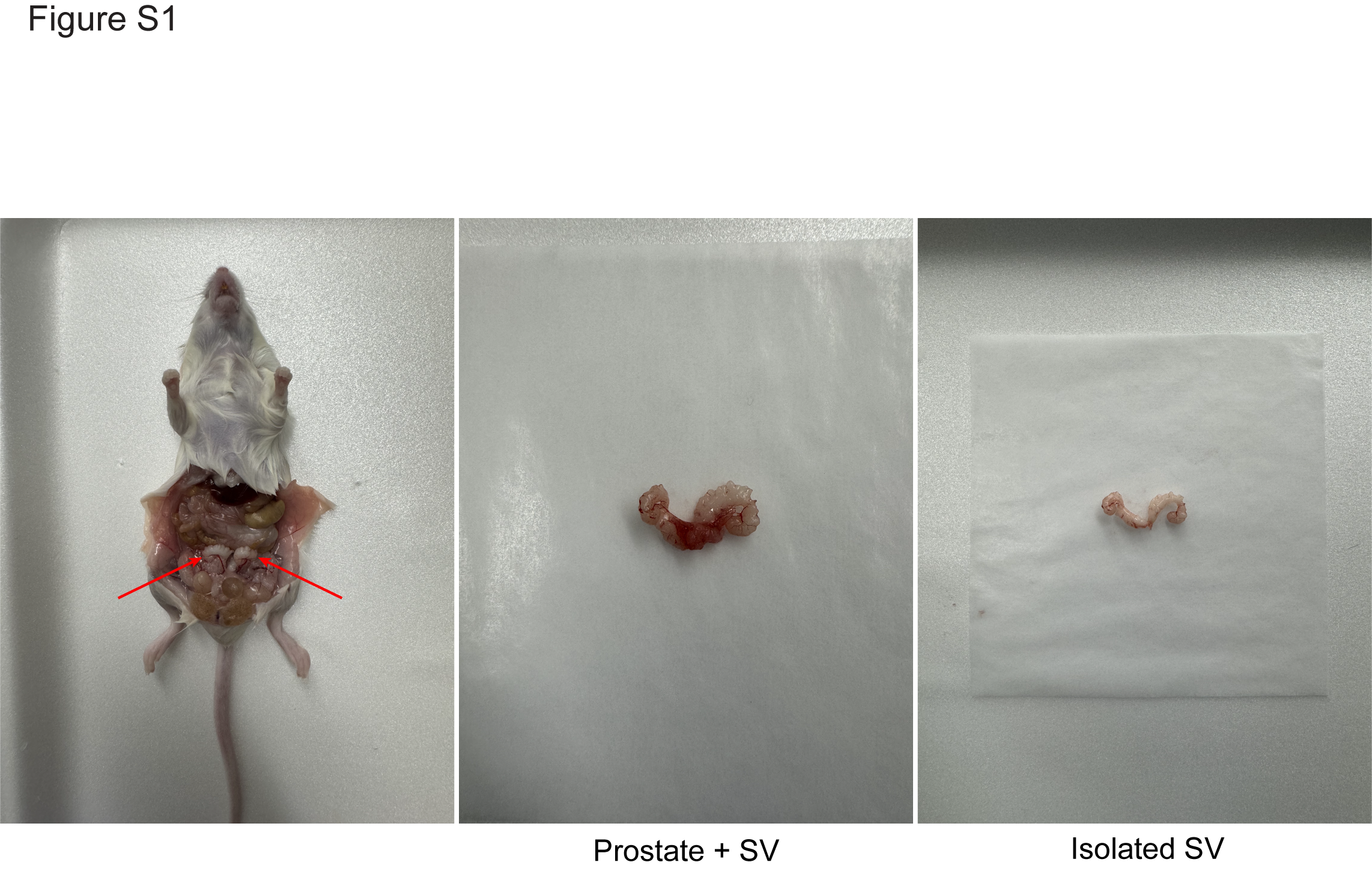

Supplement: jkaf045_Supplementary_Data [file jkaf045_supplementary_data.zip › Figure_S1_G3-2025-405724.tif]

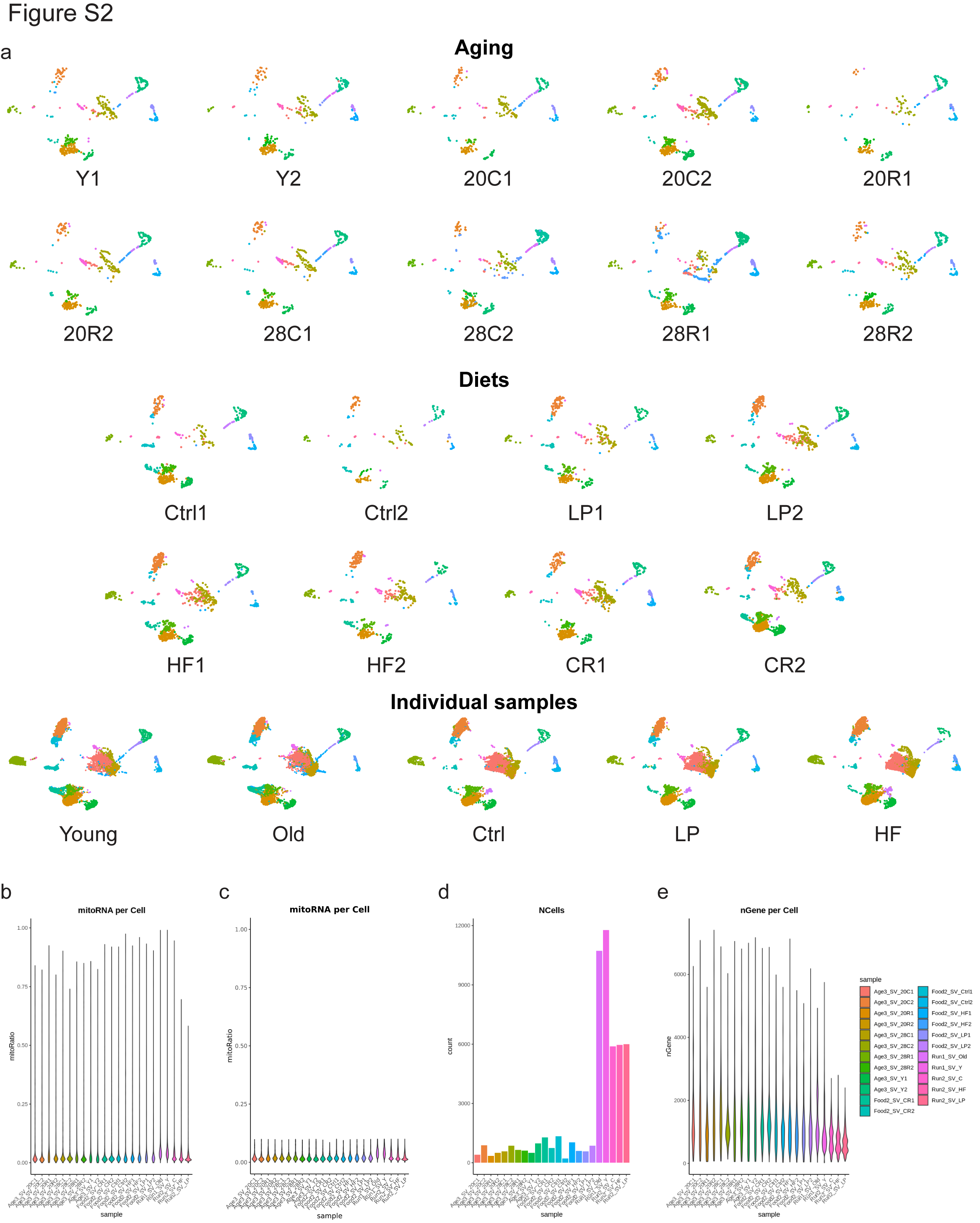

Supplement: jkaf045_Supplementary_Data [file jkaf045_supplementary_data.zip › Figure_S2_G3-2025-405724.tif]

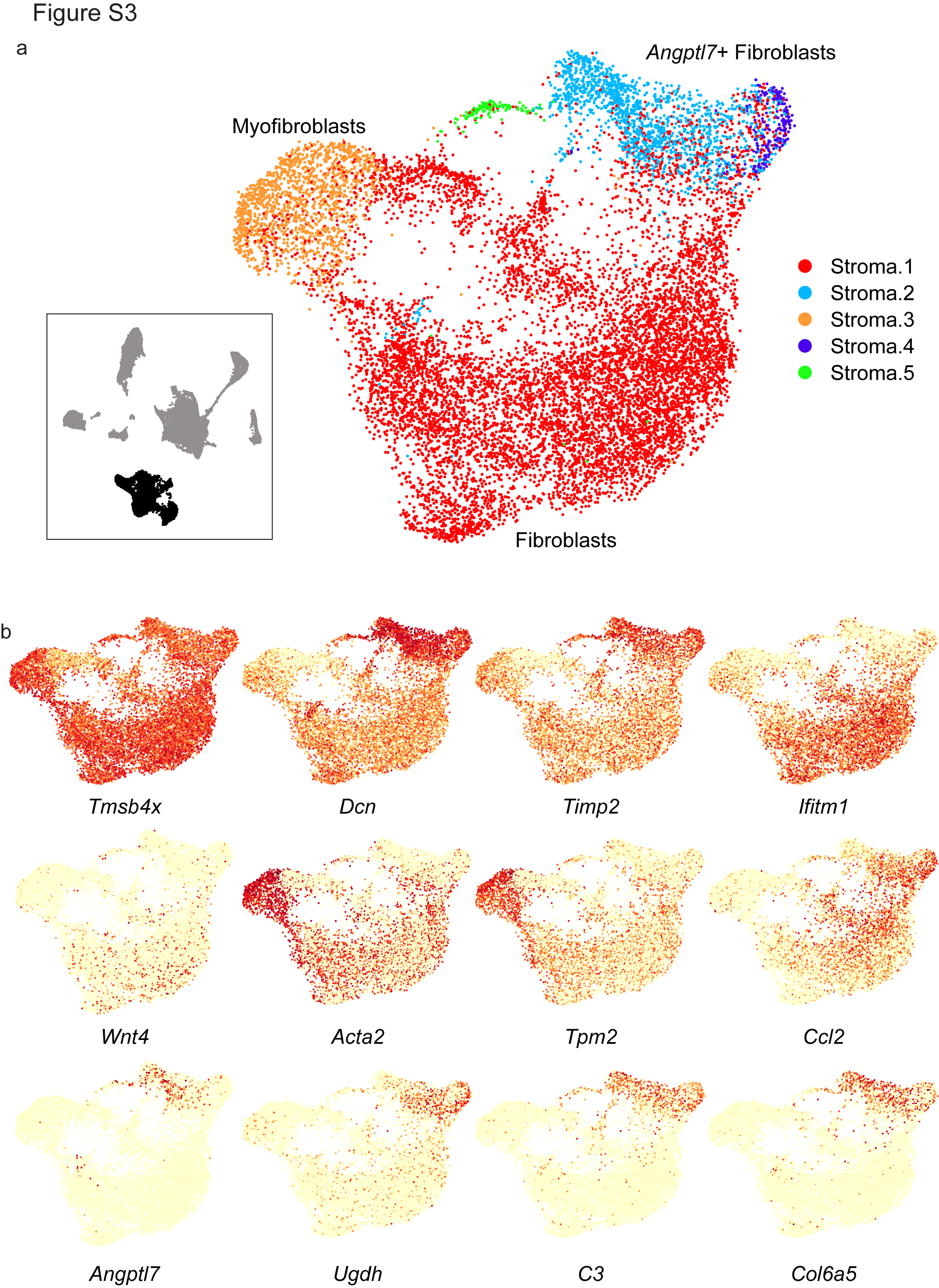

Supplement: jkaf045_Supplementary_Data [file jkaf045_supplementary_data.zip › Figure_S3_G3-2025-405724.tif]

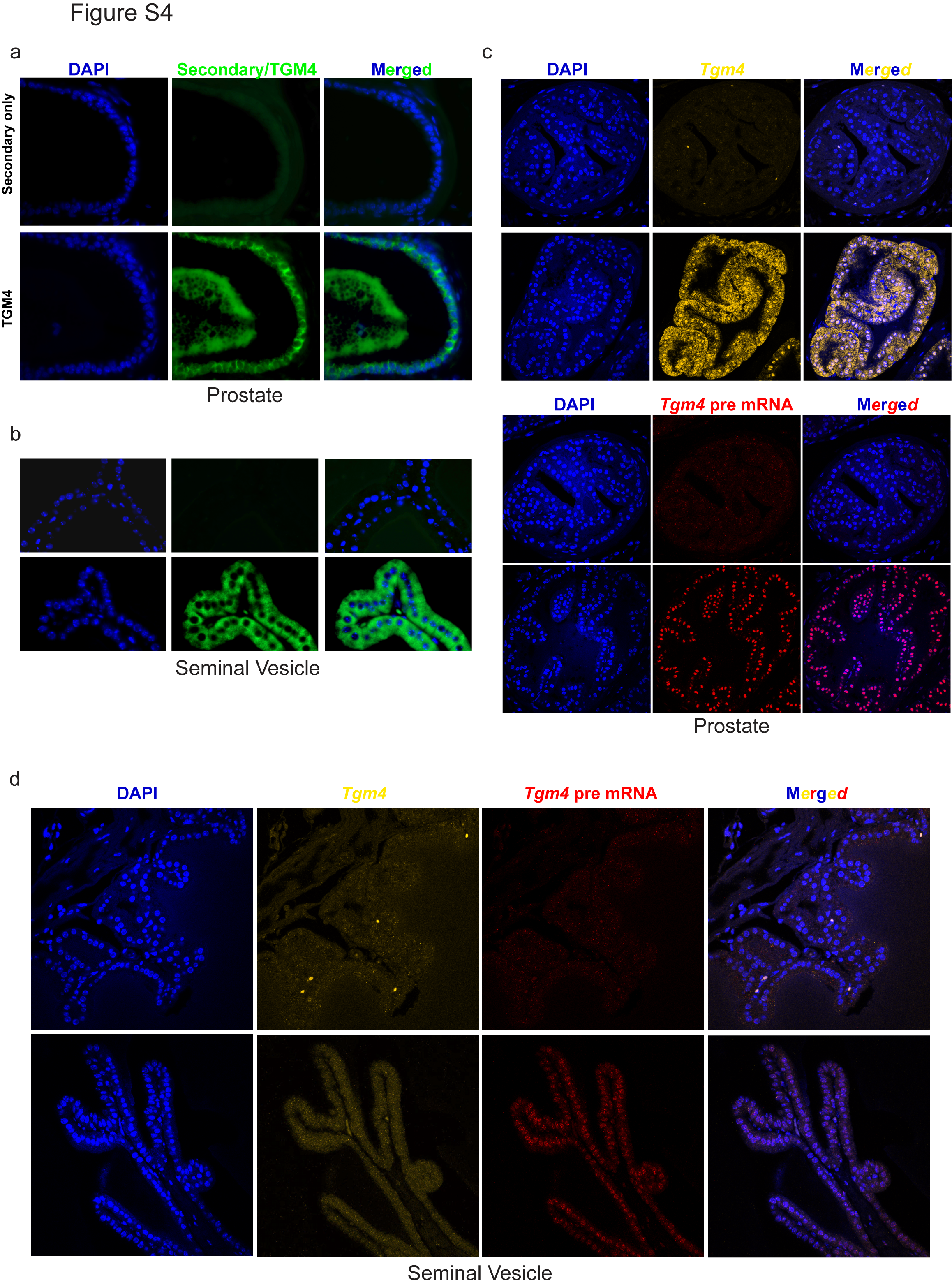

Supplement: jkaf045_Supplementary_Data [file jkaf045_supplementary_data.zip › Figure_S4_G3-2025-405724.tif]

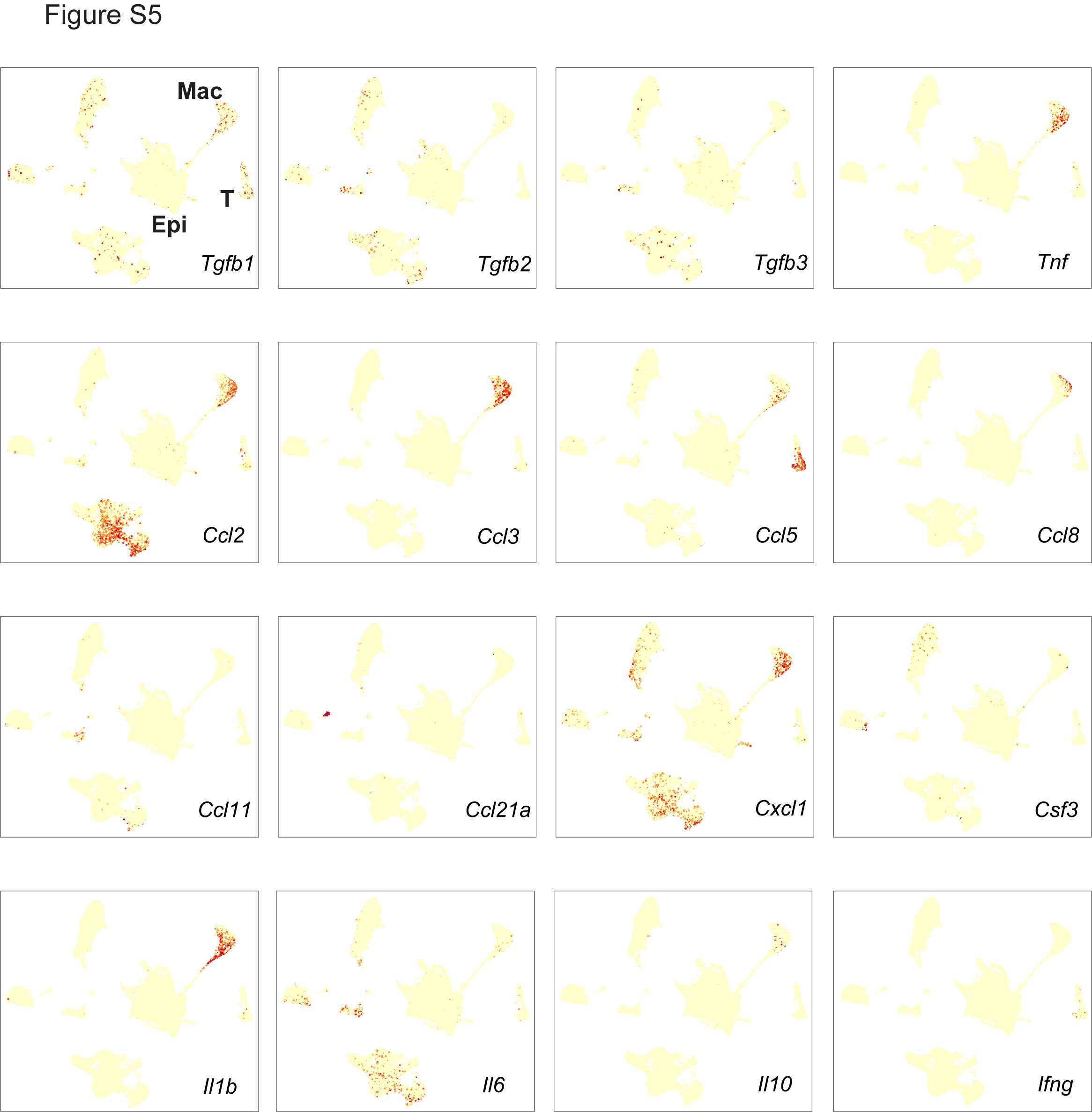

Supplement: jkaf045_Supplementary_Data [file jkaf045_supplementary_data.zip › Figure_S5_G3-2025-405724.tif]

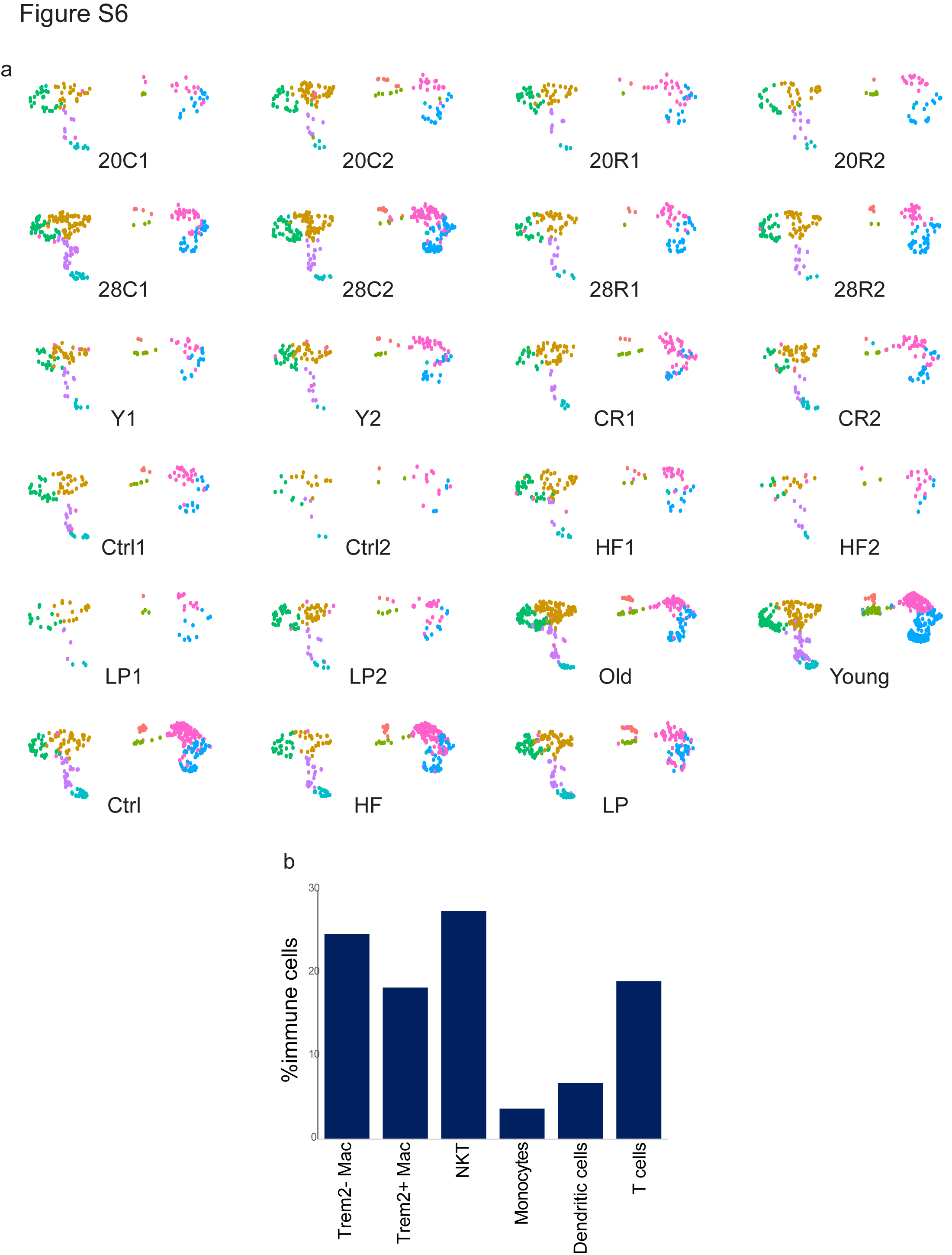

Supplement: jkaf045_Supplementary_Data [file jkaf045_supplementary_data.zip › Figure_S6_G3-2025-405724.tif]

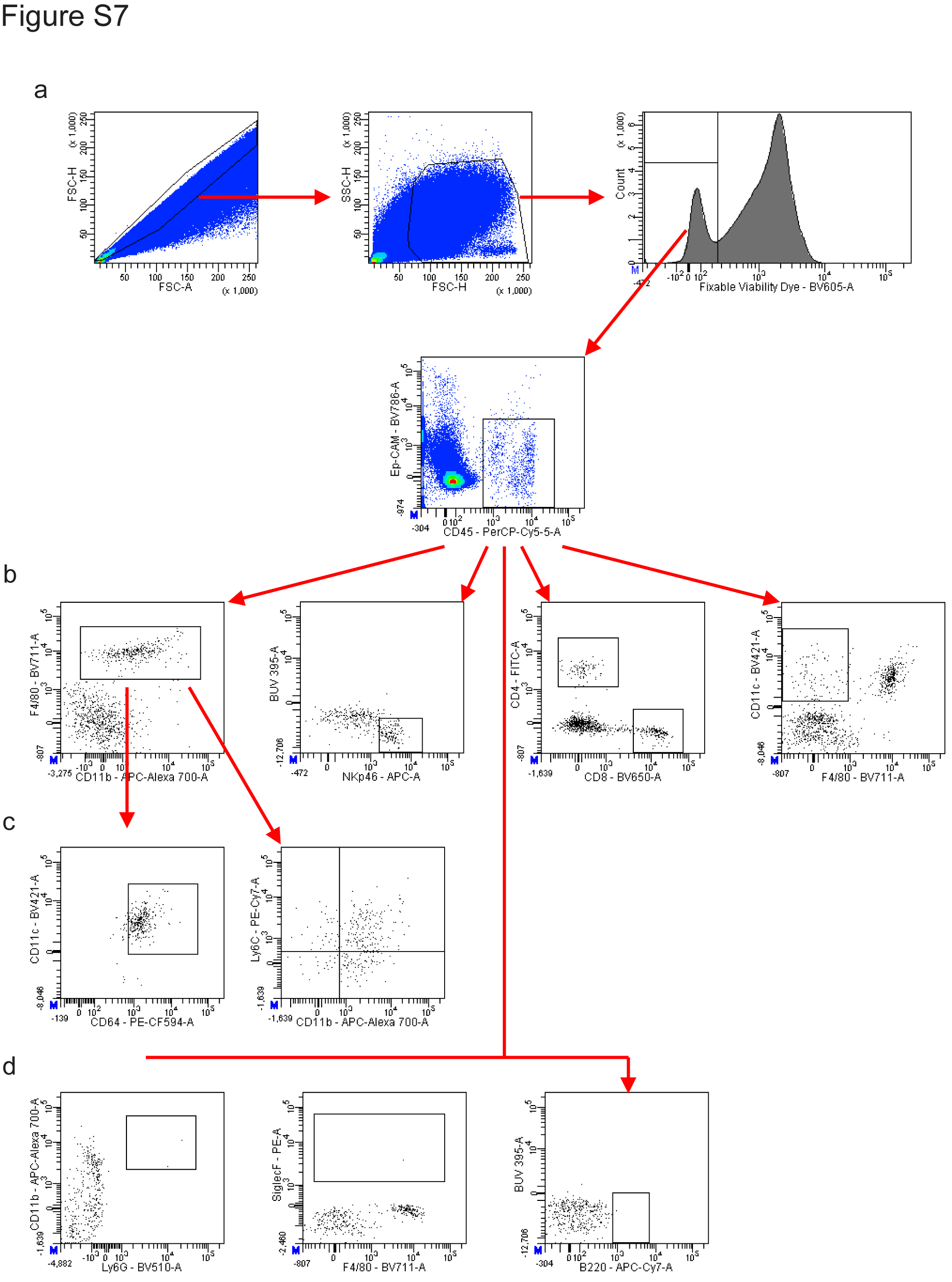

Supplement: jkaf045_Supplementary_Data [file jkaf045_supplementary_data.zip › Figure_S7_G3-2025-405724.tif]
